# Evidence of Inbreeding in Hodgkin Lymphoma

**DOI:** 10.1371/journal.pone.0154259

**Published:** 2016-04-28

**Authors:** Hauke Thomsen, Miguel Inacio da Silva Filho, Michael Fuchs, Sabine Ponader, Elke Pogge von Strandmann, Lewin Eisele, Stefan Herms, Per Hoffmann, Andreas Engert, Kari Hemminki, Asta Försti

**Affiliations:** 1 German Cancer Research Center (DKFZ), Division of Molecular Genetic Epidemiology (C050), Heidelberg, 69120, Germany; 2 Center for Primary Health Care Research, Lund University, Malmö, 20502, Sweden; 3 Department of Internal Medicine I, University Hospital of Cologne, Cologne, 50924, Germany; 4 Institute of Human Genetics and Department of Genomics, University of Bonn, Bonn, 53127, Germany; 5 Department of Biomedicine, Division of Medical Genetics, Basel, University of Basel, 4058, Switzerland; 6 Institute for Medical Informatics, Biometry and Epidemiology, University Hospital Essen, University Duisburg-Essen, Essen, 45122, Germany; IRCCS National Cancer Institute, ITALY

## Abstract

Genome-wide association studies (GWASs) have identified several, mainly co-dominantly acting, single-nucleotide polymorphisms (SNPs) associated with Hodgkin lymphoma (HL). We searched for recessively acting disease loci by performing an analysis of runs of homozygosity (ROH) based on windows of homozygous SNP-blocks and by calculating genomic inbreeding coefficients on a SNP-wise basis. We used data from a previous GWAS with 906 cases and 1217 controls from a population with a long history of no matings between relatives. Ten recurrent ROHs were identified among 25 055 ROHs across all individuals but their association with HL was not genome-wide significant. All recurrent ROHs showed significant evidence for natural selection. As a novel finding genomic inbreeding among cases was significantly higher than among controls (*P* = 2.11*10^−14^) even after correcting for covariates. Higher inbreeding among the cases was mainly based on a group of individuals with a higher average length of ROHs per person. This result suggests a correlation of higher levels of inbreeding with higher cancer incidence and might reflect the existence of recessive alleles causing HL. Genomic inbreeding may result in a higher expression of deleterious recessive genes within a population.

## Introduction

Linkage studies and genome-wide association studies (GWASs) have so far identified 8 loci to be associated with Hodgkin lymphoma (HL).[1–6] The majority of the corresponding cancer predisposition genes function in a co-dominant manner. Only a single study found a linkage consistent with a recessive inheritance on chromosome 4p, as well as on chromosomes 2, 4q, 7, 11, and 17 in 44 high-risk families for HL.[7] Population-based studies have found higher sibling risks than parent-offspring risk in HL, which suggests a presence of recessive inheritance pattern besides shared childhood exposures.[8]

Recently, a variety of studies have been performed to identify runs of homozygosity (ROHs) and to test their impact on complex diseases and traits, including cancer.[9–20] ROHs appear mainly in an increased frequency due to a high level of relatedness between individuals within a population or due to positive selection.[21] However, homozygous regions are not likely to have been selected related to cancer, which is generally of late onset relative to human life expectancy. Yet, a high level of relatedness is associated with an increased prevalence of inherited diseases. This is especially the case for recessive diseases, which only appear, if the disease allele is inherited from both parents.[22] Recessive inheritance is mainly associated with consanguinity or an increased risk in populations characterized by a higher degree of inbreeding and corresponding homozygosity.[23–27] With the development of high-density genotyping arrays, homozygosity, a component of genetic patterning, can be used to search for the cause of recessively inherited genetic diseases. Several studies have reported a significant increase in the frequency of homozygosity in cases compared with controls.[15, 16, 19, 20] However, increased homozygosity did not correlate with a higher risk of developing breast or prostate cancer or childhood B-cell precursor acute lymphoblastic leukemia (BCP-ALL).[17, 18] Even a recent study on HL did not show clear evidence of homozygosity signatures associated with HL.[28]

We conducted a whole-genome homozygosity analysis on HL based on our previous GWAS data. The aim was to examine whether homozygosity and inbreeding are associated with the risk of HL and to search for novel recessively acting disease loci.

## Material and Methods

### Genomic Data

The German HL study population comprised a total of 2 227 individuals, with 1 001 cases and 1 226 controls.[1] Cases were sampled within Germany, whereas controls were sampled within the Ruhr area in North Rhine-Westphalia as part of the Heinz-Nixdorf Recall-Study.[29] Collection of samples and clinicopathological information from subjects was undertaken with written informed consent and the Ethics committee of the University of Cologne approval in accordance with the tenets of the Declaration of Helsinki. Cases and controls were genotyped in the same laboratory using the Illumina Human OmniExpress-12 v1.0 arrays.

A detailed overview of the material including results of the GWAS study as part of the joint meta-analysis is given in our recent publication.[1] Data have been submitted to a central database: www.gwascentral.org (HGVST1823). Cases were diagnosed with HL either of mixed cellularity (132 men and 48 women; mean age at diagnosis 36.9 years, range 18–75), nodular sclerosis (211 men and 206 women; mean age at diagnosis 32.5 years, range 18–71) and further unspecified subtypes (199 men and 110 women; mean age at diagnosis 36.8 years, range 17–71). A total of 191 patients provided oral information about a positive history of infectious mononucleosis probably implicating Epstein-Barr virus infection; 547 patients denied to have had infectious mononucleosis; for 168 patients infectious mononucleosis status was unknown. No information about infectious mononucleosis in controls was available. After a stringent quality control procedure and maximizing the effective sample size, which balances the number of cases and controls best,[30] the final set consisted of 906 cases and 1 217 controls with 410 973 SNPs that had a minor allele frequency (MAF)>0.05.[31]

### Associations between homozygosity and HL

A chi^2^-test was performed to test for any association between homozygosity and susceptibility of HL on a SNP-by-SNP basis in our entire sample series.[15] To control the problem of multiple testing the false discovery rate (FDR) was calculated and controlled at an arbitrary level q* = 0.05.[32]

### Identification of runs of homozygosity

We defined ROHs following recommendations in Howrigan *et al*.[33] ROHs were detected using PLINK (v1.07) software. To prevent overestimating the number and size of ROHs no heterozygous SNPs were permitted in any window. We kept the remaining options to default values. The parameter for the “homozyg-kb” option was also kept at the default value of 1000 kb to select individual segments of minimal length. Subsequent statistical analyses were performed using packages available in the R statistics package such as “GLM”.[34] Comparison of the distribution of categorical variables was performed using the chi^2^-test in the R statistics package.[34] To compare the difference in the average number of ROHs between cases and controls, we used the Student’s t-test. Naive adjustment for multiple testing was based on the Bonferroni correction.To account for any confounding due to possible population stratification a generalized linear model was applied with 10 principle components as covariates. A permutation test based on the permutation of the regressor residuals in the R package “glmperm” was used to secure the results.[35]

### Criteria for the detection of runs of homozygosity

We used the method of Lencz *et*. *al*. to estimate the minimum number of consecutive homozygous SNPs required to form a ROH that was more than an order of magnitude larger than the mean haploblock size in the human genome without being too large to be very rare.[13] In our HL data with 2 123 individuals and 410 973 SNPs mean heterozygosity in controls was calculated to be around 35%. Therefore, a minimum length of 55 SNPs would be required to produce <5% randomly generated ROHs across all subjects ((1–0.35)^55^ x 410 973 x 2 123 = 0.04).[33] Due to linkage disequilibrium (LD) between the SNPs, the SNP genotypes are not always independent. Pairwise LD was estimated using the SNP pruning function of PLINK, with a default value of r^2^>0.8 and restricting the search of tagging SNPs within each 250 kb window. Approximately 310 000 separable tag groups were discovered, representing an >25% reduction of information compared with the original number of SNPs. Thus, ROH length of 75 was used to approximate the degrees of freedom of 55 independent SNP calls. [17]

To identify ‘common’ ROHs across the cases and the controls, or ROHs occurring only among cases or only among controls, we used packages available in R (version 3.0.2; R Foundation for Statistical Computing, Vienna, Austria). A ‘common’ ROH was defined to contain a minimum of 75 consecutive ROH calls with nearly identical start and end locations. The ‘‘homozyg-group” option of the PLINK package was used to produce a file of the overlapping ROHs separated into pools containing the number of cases and controls carrying the ROH. We considered pools with more than five samples and at least 500 kb of length as recurrent ROHs. A consensus SNP set across all samples in the pool was used to define the recurrent ROHs. Within each recurrent ROH the proportion of homozygous genotypes at each SNP was calculated for cases and controls separately, and the significance of the difference was tested by a t-test.[17] All ROH associations were robustly tested using a permutation test within the statistics package R.

### Testing the effects of natural selection

We used three metrics to investigate the selective pressure on each of the recurrent ROH. The integrated haplotype score (iHS) is based on linkage disequilibrium (LD) surrounding a positively selected allele compared with background, providing evidence of recent positive selection at a locus.[36] We also estimated F_st_ values and Fay and Wu’s H based on the frequencies of SNPs segregating in the region of interest.[37] iHS, F_st_ and Fay and Wu’s H metrics were obtained from Haplotter Software (University of Chicago, Chicago, IL, USA; http://haplotter.uchicago.edu/selection/).[36] Corresponding thresholds used were iHS >2.0, F_st_ >0.2, and Fay and Wu’s H <<-10.[36]

### Testing the effects of inbreeding

To test whether inbreeding influenced the susceptibility to HL, three different inbreeding coefficients (F I, F II and F III) were derived for each individual based on their SNP data using GCTA.[38] The coefficients were tested for differences between cases and controls using a Student’s t-test. We also used a generalized linear regression model (GLM) and regressed F I, F II or F III as explanatory variables on the disease status of the HL patient as the binary response (cases = 1/controls = 0). We included several covariates in the model: the sex of the individuals, the first 10 ancestry-informative principal components and the percentage of SNPs missing for an individual.

Finally, a genomic measure of individual homozygosity (F_ROH_) was calculated by a method similar to the one proposed by McQuillan *et al*.,[39] in which L_ROH_ is the sum of ROHs per individual above a certain criterion length (i.e. 1 000 kb as defined beforehand) and L_AUTO_ is the total SNP-mappable autosomal genome length, excluding the centromeres:

$$F_{ROH}=\sum L_{ROH}/L_{AUTO}$$

The estimated total genome length was 2 676 172 944 bp. F_ROH_ estimates inbreeding differently compared to the coefficients based on SNP-by-SNP indices F I, F II and F III as it considers only homozygous regions above a pre-defined length criterion (i.e. 1 000 kb). Based on the distribution of the F_ROH_ values in our sample we divided the data set into two subclasses with F_ROH_ values below the median and above the median.[34] The overall F_ROH_ was also tested for association with the disease status of the individuals in a GLM with the same covariates in the model as described above.

## Results

### Associations between homozygosity and HL

Initially, a test was performed for any association between homozygosity (whether for the major or the minor allele) and the susceptibility to HL on a SNP-by-SNP basis in our sample series. Results for the best SNPs with P < 1*10^−5^ are shown in the Table A in the S1 File. The most strongly associated SNP was rs11757571 [chr6: 31540765bp; chi^2^ = 22.78; P = 1.81*10^−6^]. The false discovery rate (FDR) controlled at some arbitrary level of q* did not fall below the level of q*<0.05 to indicate globally significant association.

### Association between ROHs and HL susceptibility

Within our sample set the search process for ROHs identified a total of 25 055 individual ROHs greater than 1 000 kb across all 2 123 individuals (10 479 in 906 cases and 14 576 in 1 227 controls) (Table 1). The average total length of these ROHs per person was 20 410 kb. For each individual, on average 11.80 ROH segments were detected. The average ROH size per person and the total length of ROHs per person were not different between cases and controls (Table 1), but the average number of ROHs per person was significantly lower in cases than in controls (*P* = 0.008, using a Student’s t-test and a permutation test for two independent samples).

**Table 1:** Burden analysis for cases and controls of the HL data set.

	Entire data set		
	**Cases (n = 906)**	**Controls (n = 1217)**	P*
Total number of ROHs	10479	14576	
Average number of ROHs per person	11.56	11.97	0.008
Total length of ROHs per person, kb	20122	20618	0.18
Mean ROH size per person, kb	1726	1718	0.65
	Histological subtype Mixed		
	**Cases (n = 180)**	**Controls (n = 1217)**	P*
Total number of ROHs	2085	14576	
Average number of ROHs per person	11.58	11.97	0.20
Total length of ROHs per person, kb	20230	20618	0.62
Mean ROH size per person, kb	1729	1718	0.77
	Histologial subtype Nodular sclerosis		
	**Cases (n = 417)**	**Controls (n = 1217)**	P*
Total number of ROHs	4808	14576	
Average number of ROHs per person	11.52	11.97	0.02
Total length of ROHs per person, kb	19995	20618	0.18
Mean ROH size per person, kb	1723	1718	0.80
	HL subtype–not defined		
	**Cases (n = 309)**	**Controls (n = 1217)**	P*
Total number of ROHs	3586	14576	
Average number of ROHs per person	11.60	11.97	0.10
Total length of ROHs per person, kb	20232	20618	0.46
Mean ROH size per person, kb	1728	1718	0.68
	Age subgroup cases < 42 years		
	**Cases (n = 624)**	**Controls (n = 1217)**	P*
Total number of ROHs	7194	14576	
Average number of ROHs per person	11.52	11.97	0.01
Total length of ROHs per person, kb	19869	20618	0.06
Mean ROH size per person, kb	1715	1718	0.91
	Age subgroup cases > = 42 years		
	**Cases (n = 282)**	**Controls (n = 1217)**	P*
Total number of ROHs	3285	14576	
Average number of ROHs per person	11.64	11.97	0.16
Total length of ROHs per person, kb	20684	20618	0.91
Mean ROH size per person, kb	1749	1718	0.32
	Cases–with mononucleosis		
	**Cases (n = 191)**	**Controls (n = 1217)**	P*
Total number of ROHs	2144	14576	
Average number of ROHs per person	11.22	11.97	0.004
Total length of ROHs per person, kb	19762	20618	0.20
Mean ROH size per person, kb	1746	1718	0.39
	Cases—no mononucleosis		
	**Cases (n = 547)**	**Controls (n = 1217)**	P*
Total number of ROHs	6387	14576	
Average number of ROHs per person	11.67	11.97	0.10
Total length of ROHs per person, kb	20251	20618	0.39
Mean ROH size per person, kb	1724	1718	0.77

* confirmed with a linear permutation test.

The burden analysis was extended to the histological subgroups, two different age groups and two subgroups based on self-reported information about previous infectious mononucleosis (positive/negative). In most of the subgroups the calculated parameters did not differ significantly between cases and controls. However, the average number of ROHs per person in the HL nodular sclerosis subtype was lower in cases than in controls (*P* = 0.02). The same parameter also differed significantly between cases and controls for the subgroup of patients below 42 years of age (*P* = 0.01) and for the subgroup of cases with positive history of infectious mononucleosis (*P* = 0.004). Two more subgroups were formed based on the median of the average length of ROHs per person (<1640 kb and >1640kb). Within the group of short ROHs per person (<1640 kb) the average number of ROHs per person was also significantly lower in cases than in controls (*P* = 0.009). However, among the group with long ROHs (>1640kb) the difference was not significant.

We extended the tests for association between ROHs and susceptibility to HL by categorizing the number of ROHs and the total length of ROHs in Mb (Table 2). Therefore, control groups of equal size were formed, and the numbers of cases and controls within the corresponding classes were compared. Cases had less ROHs and the total length of ROHs was also smaller than in controls. (Table 2, e.q. for entire data set >15 ROHs, OR = 0.70, *P* = 0.006; for >24.6 Mb, OR = 0.73, *P* = 0.01). A similar pattern was observed for the different subgroups, based on histology, age and self-reported history of infectious mononucleosis (Table 2). For all subgroups, cases had a lower number of ROHs and a lower total length of ROHs than controls.

**Table 2:** Association between overall ROH and HL (min. 75 SNPs per ROH).

	Entire data set				
**Number of ROH**	**Cases**	**Controls**	**OR**	**95% CI**	***P***
<10	273	294	1.00	Ref.	
10–13	299	415	0.77	0.62–0.96	**0.02**
14–15	153	233	0.70	0.54–0.91	**0.009**
>15	181	275	0.70	0.55–0.90	**0.006**
**Total Length (Mb)**					
<15.1	248	283	1.00	Ref.	
15.1–19.3	227	304	0.85	0.76–1.08	0.19
19.3–24.6	223	308	0.82	0.64–1.05	0.12
>24.6	208	322	0.73	0.57–0.94	**0.01**
	Histological subtype Mixed				
**Number of ROH**	**Cases**	**Controls**	**OR**	**95% CI**	***P***
<10	58	294	1.00	Ref.	
10–12	58	415	0.70	0.47–1.05	0.08
14–15	25	233	0.54	0.32–0.89	**0.01**
>15	39	275	0.72	0.46–1.11	0.14
**Total Length (Mb)**					
<15.1	54	283	1.00	Ref.	
15.1–19.3	46	304	0.79	0.51–1.21	0.28
19.3–24.6	33	308	0.56	0.35–0.89	**0.01**
>24.6	47	322	0.76	0.50–1.16	0.21
	Histological subtype Nodular				
**Number of ROH**	**Cases**	**Controls**	**OR**	**95% CI**	***P***
<10	126	294	1.00	Ref.	
10–13	145	415	0.81	0.61–1.08	0.15
14–15	64	233	0.64	0.45–0.90	**0.01**
>15	82	275	0.69	0.50–0.96	**0.02**
**Total Length (Mb)**					
<15.1	111	283	1.00	Ref.	
15.1–19.3	115	304	0.96	0.70–1.31	0.81
19.3–24.6	103	308	0.85	0.62–1.16	0.31
>24.6	88	322	0.69	0.50–0.96	**0.02**
	HL subtype—not defined				
**Number of ROH**	**Cases**	**Controls**	**OR**	**95% CI**	***P***
<10	89	294	1.00	Ref.	
10–13	96	415	0.76	0.55–1.05	0.10
14–15	64	233	0.90	0.62–1.30	0.60
>15	60	275	0.72	0.49–1.03	0.07
**Total Length (Mb)**					
<15.1	83	283	1.00	Ref.	
15.1–19.3	66	304	0.74	0.51–1.06	0.10
19.3–24.6	87	308	0.96	0.68–1.35	0.82
>24.6	73	322	0.77	0.54–1.10	0.15
	Age subgroup cases <42 years				
**Number of ROH**	**Cases**	**Controls**	**OR**	**95% CI**	***P***
<10	186	294	1.00	Ref.	
10–13	212	415	0.80	0.63–1.03	0.09
14–15	99	233	0.67	0.49–0.90	0.008
>15	127	275	0.73	0.55–0.96	**0.02**
**Total Length (Mb)**					
<15.1	175	283	1.00	Ref.	
15.1–19.3	158	304	0.84	0.64–1.10	0.20
19.3–24.6	152	308	0.79	0.60–1.04	0.10
>24.6	139	322	0.69	0.53–0.91	**0.01**
	Age subgroup > = 42 years				
**Number of ROH**	**Cases**	**Controls**	**OR**	**95% CI**	***P***
<10	87	294	1.00	Ref.	
10–13	87	415	0.70	0.50–0.98	**0.04**
14–15	54	233	0.78	0.53–1.14	0.20
>15	54	275	0.66	0.45–0.96	**0.03**
**Total Length (Mb)**					
<15.1	73	283	1.00	Ref.	
15.1–19.3	69	304	0.88	0.60–1.27	0.49
19.3–24.6	71	308	0.89	0.62–1.28	0.54
>24.6	69	322	0.83	0.57–1.19	0.32
	Cases–with mononucleosis				
**Number of ROH**	**Cases**	**Controls**	**OR**	**95% CI**	***P***
<10	54	294	1.00	Ref.	
10–13	82	415	1.07	0.74–1.57	0.70
14–15	26	233	0.60	0.36–0.99	**0.04**
>15	29	275	0.57	0.35–0.92	**0.02**
**Total Length (Mb)**					
<15.1	53	283	1.00	Ref.	
15.1–19.3	55	304	0.96	0.63–1.45	0.86
19.3–24.6	42	308	0.72	0.46–1.12	0.15
>24.6	41	322	0.68	0.43–1.05	0.08
	Cases with no mononucleosis				
**Number of ROH**	**Cases**	**Controls**	**OR**	**95% CI**	***P***
<10	164	294	1.00	Ref.	
10–13	170	415	0.73	0.56–0.95	**0.02**
14–15	95	233	0.73	0.53–0.99	**0.04**
>15	118	275	0.76	0.57–1.02	0.07
**Total Length (Mb)**					
<15.1	148	283	1.00	Ref.	
15.1–19.3	136	304	0.85	0.64–1.13	0.28
19.3–24.6	131	308	0.81	0.61–1.08	0.15
>24.6	132	322	0.78	0.58–1.04	0.09

For the association analysis between HL susceptibility and ROHs 4 164 consensus groups were formed, of which a total of 98 recurrent ROHs were identified in more than five samples with at least 500 kb of length and 75 SNPs. Ten recurrent ROHs were associated with HL at a suggestive level (*P*< = 0.05), but none at the genome-wide level (Table 3). Analyses were also performed for subgroups. The same recurrent ROHs were identified, but due to smaller case numbers in the subgroups recurrent ROHs were only identified in less than five samples.

**Table 3:** List of ROHs associated with HL.

ROH	Chr.	Start (bp)^a^	End (bp)^*^	Cases	Controls	Chi^2^	*P*^†^	*P*^‡^	iHS max^§^	F_st max_^||^	Fay and Wu’s H^¶^	Genes^&^
ROH1	18	25935565	26399329	7	1	6.59	0.01	0.05	2.75	0.22	-36.29	DSC3
ROH2	18	19133215	19921243	6	1	5.31	0.02	0.11	2.69	0.55	-40.42	LAMA3, **NPC1**, **RIOK3**, **C18orf8**, **ANKRD29**
ROH3	1	151925347	152835306	0	7	5.22	0.02	0.31	2.15	0.43	-33.51	CHRNB2, **IL6R**, RAB13, **RPS27**, TPM3, UBAP2L, DENND4B, HAX1, JTB, SLC27A3, C1orf43, SLC39A1, UBE2Q1, ATP8B2, GATAD2B, INTS3, AQP10, NUP210L, TDRD10, SHE, CREB3L4, MRPS33P1, CRTC2, LOC343052, C1orf189, RP11-216N14.7
ROH4	3	22624914	23524078	1	10	5.09	0.02	0.99	2.55	0.52	-35.85	LOC100129341,LOC100130785
ROH5	7	115338359	116004265	7	2	4.55	0.03	0.23	1.96	0.44	-35.50	**CAV1**, CAV2, TFEC, TES, LOC100128868
ROH6	8	30294923	31190140	5	1	4.06	0.04	8.01–03	2.42	0.62	-41.59	**GSR**, GTF2E2, PPP2CB, WRN, UBXN8, RBPMS, PURG, TEX15
ROH7	6	138307041	138849270	5	1	4.06	0.04	7.61–04	2.88	0.36	-99.25	**HEBP2**, KIAA1244, PBOV1, PERP
ROH8	5	160365553	161113298	2	11	3.98	0.04	0.98	3.38	0.38	-64.90	GABRA6, **GABRB2**, GLRXL
ROH9	9	118118896	118987836	0	5	3.73	0.05	0.59	2.81	0.43	-53.94	**TRIM32**, PAPPAS, SNORA70C
ROH10	9	23755510	24606032	1	8	3.68	0.05	0.01	3.67	0.27	-81.84	C9orf134

^*^ Chromosomal positions derived from the National Center for Biotechnology Information (NCBI), build 36, hg18

^†^ Suggestive significance, confirmed with chi^2^-permutation test performed in the statistical package R “glmperm”.

^‡^ Significances for testing differences in homozygosity with H_0_: μ_Cases_ = μ_Controls_; H_1_ (for more cases than controls): μ_Cases_ > μ_Controls_; H_1_ (for more controls than cases): μ_Cases_ < μ_Controls_

^§^ Represents maximal absolute values for iHS, derived for CEU population ancestry from Haplotter, Phase II (http://hgwen.uchicago.edu/selection/haplotter.htm)

^||^ Represents maximal values for F_st_, derived for CEU population ancestry from Haplotter, Phase II

^¶^ Represents minimum values for Fay and Wu’s H, derived for CEU population ancestry from Haplotter, Phase II (http://hgwen.uchicago.edu/selection/haplotter.htm)

^&^ Genes (bold) have been proven to be part of a network of “Origin and pathogenesis of lymphocyte-predominant Hodgkin lymphoma as revealed by global gene expression analysis” as a result of the Cancer Network Galaxy (http://tcng.hgc.jp/)

Intriguingly, several recurrent regions identified as suggestive ROHs harbor genes that have been associated with risk or progression of HL according to the Cancer Network Galaxy (http://tcng.hgc.jp/) (Table 3). The genes associated with HL have been marked in bold in Table 3. None of the ROHs encompassed the centromeric regions.

To scrutinize the significant ROH consensus regions shown in Table 3, the average homozygosity for all SNP loci within a corresponding ROH was computed separately for cases and controls and tested for a difference with a one-tailed Student’s t-test. A significant difference was observed in 3 out of 5 ROHs with more cases than controls, while ROHs with more controls than cases did not show significant differences except for ROH10 (Table 3).

### Natural selection and ROHs

ROHs have been suggested to derive from three possible mechanisms: relatedness due to demographic events (bottleneck events, founder effects or population isolation), natural selection or recent parental relatedness (inbreeding).[40] In order to assess the influence of selection on the most promising ROH regions, three estimates were used iHS, F_st_ and Fay and Wu’s H.[36, 41, 42] Every ROH of interest showed significant values for the three estimates (iHS >2.0, F_st_ >0.2 and Fay and Wu’s H <<-10; Table 3), indicating that each of the ten ROH regions might be the result of a selective sweep.

### Inbreeding and HL

**Fig 1:**
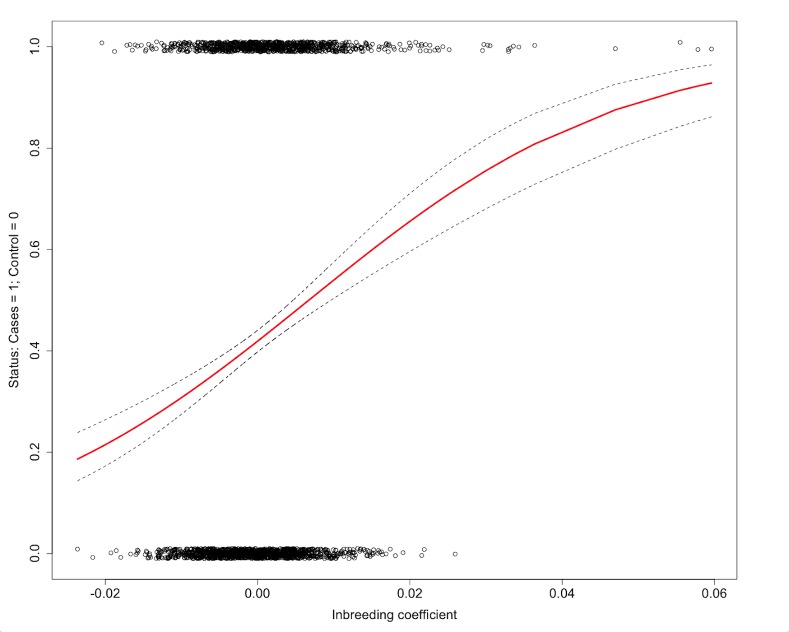
Regression slope of inbreeding coefficient F III on disease status (including confidence interval). The inbreeding coefficient F III as a continuous variable is used in a generalized linear model as an explanatory variable on the disease status of the study participants defined as the binary response (0/1).

We formally calculated the inbreeding coefficients (so called F I, F II and F III) after Yang *et al*. for all samples in the set.[38] F I is based on the variance of additive genetic values, F II on SNP homozygosity and F III on the correlation between uniting gametes. The means (SDs) for F I in cases and controls were 0.002 (0.008) and -0.0005 (0.006), respectively, and significantly different from each other (*P* = 2.11*10^−14^) by a Student’s t-test including a permutation test and by regression of the explanatory variable F I on the disease status of the HL patient as a binary response (cases = 1/controls = 0) in a GLM with no covariates in the model. Thus, there was significant evidence that cases were in general more inbred than controls. This was supported by the inbreeding coefficients F II and F III, which also differed significantly between cases and controls at *P* = 0.009 and *P* = 3.37*10^−5^, with cases being more inbred. Fig 1 illustrates the results of a GLM with no covariates in the model. The explanatory inbreeding coefficient F III as a continuous variable is regressed on the disease status of the cases and controls defined as a binary response (cases = 1/controls = 0). It also shows the regression line and the corresponding confidence bands. Since the response variable is discrete some jitter was added to minimize overlap among the case group or control group. The slope of the regression line clearly increases with an increasing inbreeding coefficient tending towards the affected individuals.

We extended the GLM by including several covariates to test the effect of the explanatory inbreeding coefficients, F I, F II, and F III, on the disease status. Both F I and F III remained significant at *P* = 0.02 with a positive effect estimate varying from 17.25 to 34.49, which resulted in an increasing slope of the regression line towards the diseased individuals (data not shown). F II was not significant at *P* = 0.05, however the trend was similar.

The same analysis was performed on both subgroups that were derived based on the median of the average length of ROHs per person (<1640 kb and >1640kb). Within the group of long ROHs per person (>1640 kb) the inbreeding coefficients FI, FII and FIII were significantly higher in cases than in controls (*P* = 0.004). However, among the group with shorter ROHs (<1640kb) the difference was not significant.

Inbreeding coefficients FI, FII and FIII were also checked for differences of the different age and histological subgroups against controls and for the subgroups of cases with positive or negative history of infectious mononucleosis against controls. Differences were not significant.

**Fig 2:**
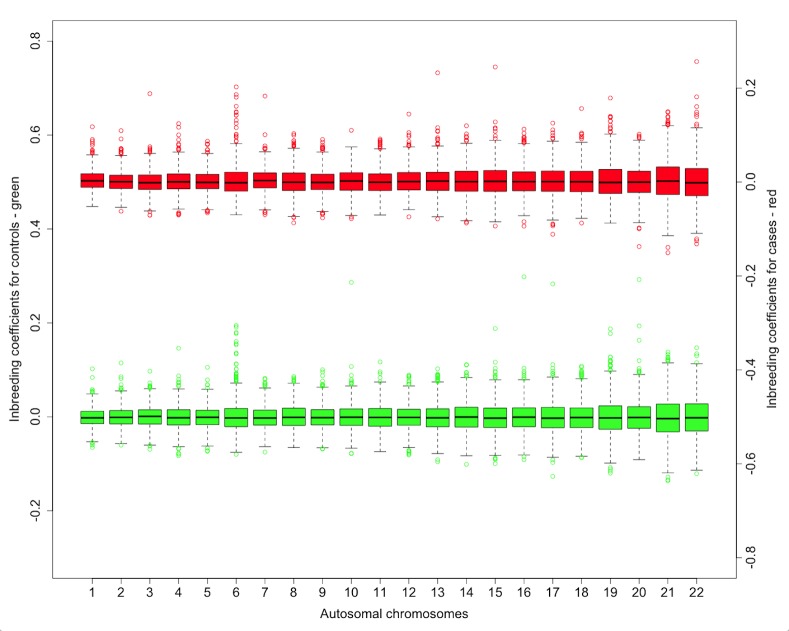
Variation of inbreeding coefficient among chromosomes. The boxplot figure shows the means and variation of the inbreeding coefficient F I for autosomal chromosomes 1 to 22 for cases (red–with right-handed ordinate) and controls (green–with left-handed ordinate).

In Fig 2 the variation of the inbreeding coefficient between chromosomes is shown. The mean is rather constant across the chromosomes but the variation is increasing from chromosome 1 to 22 while the length of the chromosomes in base pairs is decreasing (r = -0.81, P = 3.30*10^−6^). Fig 2 points out that several individuals are more inbred for chromosome 6 compared to other chromosomes. Closer investigation of chromosome 6 showed that the mean was significantly higher in cases (0.003, SD 0.02) than in controls (0.0001, SD 0.02) with a *P*-value of 0.001. This difference remained significant with a *P*-value of 0.008, even after exclusion of the entire HLA region, the strongest genetic risk region of HL. A similar pattern was observed for chromosomes 1, 7, and 13 with corresponding *P*-values of 0.003, 0.03 and 0.10, respectively.

Three additional associations for different inbreeding measures were tested. Results are shown in Fig A in the S1 File. The total length of individual ROHs is highly correlated with the total number of ROHs per individual (r = 0.77, *P*< 2.20*10^−16^). A moderate association is determined for the total length of ROHs per individual and the individual inbreeding coefficient F III (r = 0.36, *P*< 2.20*10^−16^), while the lowest association was determined for the total number of ROHs per individual and the individual inbreeding coefficient F III (r = 0.25, *P*< 2.20*10^−16^).

Finally, we checked the association between homozygosity and the susceptibility to HL by comparing the number of cases against equally distributed numbers of controls for different F_ROH_ values (Table B in S1 File). The ratio of cases vs. controls is decreasing with an increase of F_ROH_. Odds ratios and corresponding P values are significant for the highest class of F_ROH_. The pattern was similar for short ROHs but was not seen for the long ROHs. As F_ROH_ is deemed to represent a function of the total length of ROHs of each individual’s genome, we also tested the correlation between the inbreeding coefficients F I, F II and F III and F_ROH_, which were rather moderate (r_FI_ = 0.35, *P* = 2.20*10^−16^, r_FII_ = 0.34, *P* = 2.20*10^−16^, r_FIII_ = 0.36, *P* = 2.20*10^−16^). Testing F_ROH_ for an association with the disease status of the individuals in a GLM with the covariates in the model did not show F_ROH_ to have a significant effect (*P* = 0.66).

## Discussion

The current work is to our knowledge the first analysis of the influence of inbreeding on the susceptibility to HL. Our study was based on a prior GWAS that successfully identified two novel germline variants associated with HL.[1]

Homozygosity can be caused by demographic events, consanguinity/inbreeding or selective pressure.[43]^,^[44] Most of the ROHs in our study were relatively short. This excludes recent consanguinity as the cause of inbreeding. However, inbreeding coefficients still point to a certain level of relatedness that might remain from distant consanguinity. All ROHs of interest showed significant evidence for natural selection (iHS, F_st_, Fay and Wu’s H).[36] The influence of selective pressure on the ROH length therefore, cannot be excluded.

We did not discover any genome-wide association between homozygosity and susceptibility to HL on a SNP-by-SNP basis. Further downstream analyses revealed differences between cases and controls in terms of the number of ROHs per person. In contrast to previous studies in other cancers, which either reported increased frequency of homozygosity in cases or did not find any differences between cases and controls,[15–20, 28],[43] the average number of ROHs per person in our material was significantly lower among cases, although the extent was small. The main differences between cases and controls in terms of the number of ROHs per person arise from the group of individuals with shorter ROHs per person, whereas differences between cases and controls among individuals with longer ROHs per person were not significant and, therefore, exclude recent consanguinity. This is supported by the analyses for the different age and histological subgroups and for the subgroups of cases with positive and negative history of infectious mononucleosis.

The analysis of recurrent ROHs did not result in genome-wide significant associations with HL, and there was no clear pattern about overlapping ROH regions being absent or present in cases solely. Three of the ROHs identified in our analysis (ROH1, ROH6 and ROH8) overlap with long contiguous stretches of homozygosity from studies that were aimed to detect differences between human outbred populations.[45, 46] The previously suggested recessively acting HL loci on chromosomes 2, 4, 7, 11 and 17 were not confirmed in our study,[7] nor did any of our recurrent ROHs overlap with ROHs detected recently in HL.[28] With respect to these studies we conclude, that there is no absolute evidence of an association between extended stretches of homozygosity and an increased HL risk. This result is not unexpected as several even more powerful studies published earlier did not detect any remarkable association between ROHs and cancer susceptibility.[17, 18, 43]

The novel result of our study is the significant effect of genomic inbreeding among cases and its possible effect on the development of the disease. The inbreeding coefficients F I, F II and F III were significantly higher in cases than in controls, and the coefficients F I and F III remained significantly higher among cases after correcting for covariates using GLM including a permutation test. These results seem to be opposite to the ROH analysis, in which controls had more ROHs than cases. This is, because inbreeding coefficients are calculated on a SNP-wise basis, whereas a ROH spans a window of homozygous SNP-blocks. In fact, within the subgroup of individuals with shorter sizes of ROHs per person the inbreeding coefficients F I, F II and F III did not differ significantly. However, among the other subgroup with longer sizes of ROHs per person inbreeding coefficients F I, F II and F III were significantly higher in cases even after correction for covariates.

With a higher level of inbreeding chances being affected by recessive or deleterious traits are increased.[47] In fact, the assumption that a higher level of inbreeding correlates with cancer incidence has been proven already on the population level and on genomic level.[19, 48],[49] Compared to the inbreeding coefficients F I, F II and F III, which are SNP-by-SNP-based, F_ROH_ represents a function of the total length of ROHs in each individual. Yet, the F_ROH_ is discarding SNPs below our minimum ROH length criterion of 1 000 kb. The fact, that we found small but significant differences among cases and controls in the mean sum of shorter ROHs but not for the long ROHs also supports the view that the differences in ROHs length shorter than 1.6Mb reflect LD pattern of ancient origin rather than effects of more recent inbreeding.[39] Although some of the long ROHs probably reflect recent parental relatedness, most of them potentially result from a lack of recombination that allows unusually long ancestral segments to persist in the general population with a low pressure of recombination equally distributed to cases and controls.[39] Overall, the current approaches to the computation of homozygosity assume that the founders of the pedigrees are unrelated, and this assumption is realistic because both cases and controls are from a population with a long history of no matings between relatives. Therefore, in our outbred population shorter thresholds are optimal for detecting significant homozygosity.[50]

In conclusion, ten recurrent ROHs were identified. All recurrent ROHs showed significant evidence for natural selection. Higher inbreeding among cases may suggest the existence of recessive alleles that cause HL. Inbreeding can result in a higher phenotypic expression of deleterious recessive genes within a population. The genetic architecture of HL is therefore most likely consistent with a genetic model, in which the genetic variants are more likely to be rare than common. However, they are also likely to be numerous with highly polygenic architecture and of a small individual effect. If this view on the genetic architecture of HL were correct, it would be important to consider inbreeding as an influence on the disease.

## Supporting Information

S1 File Combined Supporting Information File. **Table A,** Association between homozygosity and susceptibility to HL for individual SNPs. **Table B,** Association between F_ROH_ and HL. **Fig A,** Pearson's correlation coefficients for different consanguinity measures. **Fig B,** ROHs on chromosome 18.
(PDF)

## References

[1] Frampton M, da Silva Filho MI, Broderick P, Thomsen H, Forsti A, Vijayakrishnan J, et al Variation at 3p24.1 and 6q23.3 influences the risk of Hodgkin's lymphoma. Nature communications. 2013;4:2549 Epub 2013/10/24. 10.1038/ncomms3549 . 24149102 PMC5053363

[2] Enciso-Mora V, Broderick P, Ma Y, Jarrett RF, Hjalgrim H, Hemminki K, et al A genome-wide association study of Hodgkin's lymphoma identifies new susceptibility loci at 2p16.1 (REL), 8q24.21 and 10p14 (GATA3). Nature Genetics. 2010;42(12):1126–30. 10.1038/ng.696 21037568 PMC4268499

[3] Urayama KY, Jarrett RF, Hjalgrim H, Diepstra A, Kamatani Y, Chabrier A, et al Genome-Wide Association Study of Classical Hodgkin Lymphoma and Epstein-Barr Virus Status-Defined Subgroups. JNCI Journal of the National Cancer Institute. 2012;104(3):240–53. 10.1093/jnci/djr516 22286212 PMC3274508

[4] Cozen W, Li D, Best T, Van Den Berg DJ, Gourraud PA, Cortessis VK, et al A genome-wide meta-analysis of nodular sclerosing Hodgkin lymphoma identifies risk loci at 6p21.32. Blood. 2012;119(2):469–75. 10.1182/blood-2011-03-343921 22086417 PMC3257012

[5] Cozen W, Timofeeva MN, Li D, Diepstra A, Hazelett D, Delahaye-Sourdeix M, et al A meta-analysis of Hodgkin lymphoma reveals 19p13.3 TCF3 as a novel susceptibility locus. Nature communications. 2014;5:3856 10.1038/ncomms4856 24920014 PMC4055950

[6] Sud A, Hemminki K, Houlston RS. Candidate gene association studies and risk of Hodgkin lymphoma: a systematic review and meta-analysis. Hematol Oncol. 2015 10.1002/hon.2235 . 26053036 PMC6175040

[7] Goldin LR, McMaster ML, Ter-Minassian M, Saddlemire S, Harmsen B, Lalonde G, et al A genome screen of families at high risk for Hodgkin lymphoma: evidence for a susceptibility gene on chromosome 4. Journal of medical genetics. 2005;42(7):595–601. Epub 2005/07/05. 10.1136/jmg.2004.027433 15994882 PMC1736088

[8] Hemminki K, Sundquist J, Lorenzo Bermejo J. Familial risks for cancer as the basis for evidence-based clinical referral and counseling. The oncologist. 2008;13(3):239–47. 10.1634/theoncologist.2007-0242 . 18378534

[9] Mok K, Laaksovirta H, Tienari PJ, Peuralinna T, Myllykangas L, Chio A, et al Homozygosity analysis in amyotrophic lateral sclerosis. European journal of human genetics: EJHG. 2013;21(12):1429–35. Epub 2013/04/25. 10.1038/ejhg.2013.59 23612577 PMC3829775

[10] Ghani M, Sato C, Lee JH, Reitz C, Moreno D, Mayeux R, et al Evidence of Recessive Alzheimer Disease Loci in a Caribbean Hispanic Data Set: Genome-wide Survey of Runs of Homozygosity. JAMA neurology. 2013 Epub 2013/08/28. 10.1001/jamaneurol.2013.3545 . 23978990 PMC3991012

[11] Yang TL, Guo Y, Zhang LS, Tian Q, Yan H, Papasian CJ, et al Runs of homozygosity identify a recessive locus 12q21.31 for human adult height. The Journal of clinical endocrinology and metabolism. 2010;95(8):3777–82. Epub 2010/05/15. 10.1210/jc.2009-1715 20466785 PMC2913044

[12] Nalls MA, Guerreiro RJ, Simon-Sanchez J, Bras JT, Traynor BJ, Gibbs JR, et al Extended tracts of homozygosity identify novel candidate genes associated with late-onset Alzheimer's disease. Neurogenetics. 2009;10(3):183–90. Epub 2009/03/10. 10.1007/s10048-009-0182-4 19271249 PMC2908484

[13] Lencz T, Lambert C, DeRosse P, Burdick KE, Morgan TV, Kane JM, et al Runs of homozygosity reveal highly penetrant recessive loci in schizophrenia. Proceedings of the National Academy of Sciences of the United States of America. 2007;104(50):19942–7. Epub 2007/12/14. 10.1073/pnas.0710021104 18077426 PMC2148402

[14] Gamsiz ED, Viscidi EW, Frederick AM, Nagpal S, Sanders SJ, Murtha MT, et al Intellectual disability is associated with increased runs of homozygosity in simplex autism. American journal of human genetics. 2013;93(1):103–9. Epub 2013/07/09. 10.1016/j.ajhg.2013.06.004 23830515 PMC3710760

[15] Spain SL, Cazier JB, Consortium C, Houlston R, Carvajal-Carmona L, Tomlinson I. Colorectal cancer risk is not associated with increased levels of homozygosity in a population from the United Kingdom. Cancer research. 2009;69(18):7422–9. Epub 2009/09/03. 10.1158/0008-5472.CAN-09-0659 . 19723657

[16] Bacolod MD, Schemmann GS, Wang S, Shattock R, Giardina SF, Zeng Z, et al The signatures of autozygosity among patients with colorectal cancer. Cancer research. 2008;68(8):2610–21. Epub 2008/04/01. 10.1158/0008-5472.CAN-07-5250 . 18375840 PMC4383032

[17] Enciso-Mora V, Hosking FJ, Houlston RS. Risk of breast and prostate cancer is not associated with increased homozygosity in outbred populations. European journal of human genetics: EJHG. 2010;18(8):909–14. Epub 2010/04/22. 10.1038/ejhg.2010.53 20407466 PMC2987391

[18] Hosking FJ, Papaemmanuil E, Sheridan E, Kinsey SE, Lightfoot T, Roman E, et al Genome-wide homozygosity signatures and childhood acute lymphoblastic leukemia risk. Blood. 2010;115(22):4472–7. Epub 2010/03/17. 10.1182/blood-2009-09-244483 . 20231427

[19] Wang C, Xu Z, Jin G, Hu Z, Dai J, Ma H, et al Genome-wide analysis of runs of homozygosity identifies new susceptibility regions of lung cancer in Han Chinese. Journal of biomedical research. 2013;27(3):208–14. Epub 2013/05/31. 10.7555/JBR.27.20130017 23720676 PMC3664727

[20] Assie G, LaFramboise T, Platzer P, Eng C. Frequency of germline genomic homozygosity associated with cancer cases. JAMA: the journal of the American Medical Association. 2008;299(12):1437–45. Epub 2008/03/28. 10.1001/jama.299.12.1437 . 18364486

[21] Kijas JW. Detecting regions of homozygosity to map the cause of recessively inherited disease. Methods in molecular biology. 2013;1019:331–45. Epub 2013/06/13. 10.1007/978-1-62703-447-0_14 . 23756898

[22] Lander ES, Botstein D. Homozygosity mapping: a way to map human recessive traits with the DNA of inbred children. Science. 1987;236(4808):1567–70. Epub 1987/06/19. . 2884728 10.1126/science.2884728

[23] Abramson JH, Pridan H, Sacks MI, Avitzour M, Peritz E. A case-control study of Hodgkin's disease in Israel. Journal of the National Cancer Institute. 1978;61(2):307–14. Epub 1978/08/01. . 277717

[24] Bener A, El Ayoubi HR, Chouchane L, Ali AI, Al-Kubaisi A, Al-Sulaiti H, et al Impact of consanguinity on cancer in a highly endogamous population. Asian Pacific journal of cancer prevention: APJCP. 2009;10(1):35–40. Epub 2009/05/28. . 19469621

[25] Feldman JG, Lee SL, Seligman B. Occurrence of acute leukemia in females in a genetically isolated population. Cancer. 1976;38(6):2548–50. Epub 1976/12/01. . 1069604 10.1002/1097-0142(197612)38:6<2548::aid-cncr2820380644>3.0.co;2-y

[26] Lebel RR, Gallagher WB. Wisconsin consanguinity studies. II: Familial adenocarcinomatosis. American journal of medical genetics. 1989;33(1):1–6. Epub 1989/05/01. 10.1002/ajmg.1320330102 . 2750776

[27] Shami SA, Qaisar R, Bittles AH. Consanguinity and adult morbidity in Pakistan. Lancet. 1991;338(8772):954 Epub 1991/10/12. . 1681304 10.1016/0140-6736(91)91828-i

[28] Sud A, Cooke R, Swerdlow AJ, Houlston RS. Genome-wide homozygosity signature and risk of Hodgkin lymphoma. Sci Rep. 2015;5:14315 10.1038/srep14315 26391888 PMC4585760

[29] Schmermund A, Mohlenkamp S, Stang A, Gronemeyer D, Seibel R, Hirche H, et al Assessment of clinically silent atherosclerotic disease and established and novel risk factors for predicting myocardial infarction and cardiac death in healthy middle-aged subjects: rationale and design of the Heinz Nixdorf RECALL Study. Risk Factors, Evaluation of Coronary Calcium and Lifestyle. American heart journal. 2002;144(2):212–8. . 12177636 10.1067/mhj.2002.123579

[30] Winkler TW, Day FR, Croteau-Chonka DC, Wood AR, Locke AE, Magi R, et al Quality control and conduct of genome-wide association meta-analyses. Nat Protoc. 2014;9(5):1192–212. 10.1038/nprot.2014.071 24762786 PMC4083217

[31] Thomsen H, da Silva Filho MI, Forsti A, Fuchs M, Ponader S, von Strandmann EP, et al Heritability estimates on Hodgkin's lymphoma: a genomic- versus population-based approach. European journal of human genetics: EJHG. 2014 10.1038/ejhg.2014.184 . 25227146 PMC4795060

[32] Weller JI, Song JZ, Heyen DW, Lewin HA, Ron M. A new approach to the problem of multiple comparisons in the genetic dissection of complex traits. Genetics. 1998;150(4):1699–706. 9832544 10.1093/genetics/150.4.1699 PMC1460417

[33] Howrigan DP, Simonson MA, Keller MC. Detecting autozygosity through runs of homozygosity: a comparison of three autozygosity detection algorithms. BMC genomics. 2011;12:460 Epub 2011/09/29. 10.1186/1471-2164-12-460 21943305 PMC3188534

[34] Team RC. R: A Language and Environment for Statistical Computing. 2013.

[35] Werft W, Benner A. glmperm: A Permutation of Regressor Residuals Test for Inference in Generalized Linear Models. R J. 2010;2(1):39–43. .

[36] Voight BF, Kudaravalli S, Wen X, Pritchard JK. A map of recent positive selection in the human genome. PLoS biology. 2006;4(3):e72 Epub 2006/02/24. 10.1371/journal.pbio.0040072 16494531 PMC1382018

[37] Fay JC, Wu CI. Hitchhiking under positive Darwinian selection. Genetics. 2000;155(3):1405–13. Epub 2000/07/06. 10880498 10.1093/genetics/155.3.1405 PMC1461156

[38] Yang J, Lee SH, Goddard ME, Visscher PM. GCTA: a tool for genome-wide complex trait analysis. American journal of human genetics. 2011;88(1):76–82. 10.1016/j.ajhg.2010.11.011 21167468 PMC3014363

[39] McQuillan R, Leutenegger AL, Abdel-Rahman R, Franklin CS, Pericic M, Barac-Lauc L, et al Runs of homozygosity in European populations. American journal of human genetics. 2008;83(3):359–72. Epub 2008/09/02. 10.1016/j.ajhg.2008.08.007 18760389 PMC2556426

[40] Pemberton TJ, Absher D, Feldman MW, Myers RM, Rosenberg NA, Li JZ. Genomic patterns of homozygosity in worldwide human populations. American journal of human genetics. 2012;91(2):275–92. Epub 2012/08/14. 10.1016/j.ajhg.2012.06.014 22883143 PMC3415543

[41] Coop G, Pickrell JK, Novembre J, Kudaravalli S, Li J, Absher D, et al The role of geography in human adaptation. PLoS Genet. 2009;5(6):e1000500 10.1371/journal.pgen.1000500 19503611 PMC2685456

[42] Oleksyk TK, Smith MW, O'Brien SJ. Genome-wide scans for footprints of natural selection. Philosophical transactions of the Royal Society of London Series B, Biological sciences. 2010;365(1537):185–205. 10.1098/rstb.2009.0219 20008396 PMC2842710

[43] Siraj AK, Khalak HG, Sultana M, Al-Rasheed M, Bavi P, Al-Sanea N, et al Colorectal cancer risk is not associated with increased levels of homozygosity in Saudi Arabia. Genetics in medicine: official journal of the American College of Medical Genetics. 2012 Epub 2012/04/07. 10.1038/gim.2012.27 . 22481135

[44] Woods CG, Cox J, Springell K, Hampshire DJ, Mohamed MD, McKibbin M, et al Quantification of homozygosity in consanguineous individuals with autosomal recessive disease. American journal of human genetics. 2006;78(5):889–96. Epub 2006/04/28. 10.1086/503875 16642444 PMC1474039

[45] Li LH, Ho SF, Chen CH, Wei CY, Wong WC, Li LY, et al Long contiguous stretches of homozygosity in the human genome. Hum Mutat. 2006;27(11):1115–21. Epub 2006/09/07. 10.1002/humu.20399 . 16955415

[46] Gibson J, Morton NE, Collins A. Extended tracts of homozygosity in outbred human populations. Human molecular genetics. 2006;15(5):789–95. Epub 2006/01/27. 10.1093/hmg/ddi493 . 16436455

[47] Nabulsi MM, Tamim H, Sabbagh M, Obeid MY, Yunis KA, Bitar FF. Parental consanguinity and congenital heart malformations in a developing country. American journal of medical genetics Part A. 2003;116A(4):342–7. 10.1002/ajmg.a.10020 . 12522788

[48] Rudan I. Inbreeding and cancer incidence in human isolates. Human biology. 1999;71(2):173–87. Epub 1999/05/01. . 10222641

[49] Spielman D, Brook BW, Briscoe DA, Frankham R. Does inbreeding and loss of genetic diversity decrease disease resistance? Conserv Genet. 2004;5(4):439–48. 10.1023/B:Coge.0000041030.76598.Cd .

[50] Keller MC, Visscher PM, Goddard ME. Quantification of inbreeding due to distant ancestors and its detection using dense single nucleotide polymorphism data. Genetics. 2011;189(1):237–49. Epub 2011/06/28. 10.1534/genetics.111.130922 21705750 PMC3176119

