# Supplementary material for: Evidence of Inbreeding in Hodgkin Lymphoma
Source: PLoS One. 2016 Apr 28;11(4):e0154259. doi: 10.1371/journal.pone.0154259 (PMC4849743; doi:10.1371/journal.pone.0154259)
Supplement: S1 File — Table A, Association between homozygosity and susceptibility to HL for individual SNPs. Table B, Association between FROH and HL. Fig A, Pearson's correlation coefficients for different consanguinity measures. Fig B, ROHs on chromosome 18. (PDF) [file pone.0154259.s001.pdf]

Evidence of inbreeding in Hodgkin lymphoma

Supplementary Material

Hauke Thomsen[1]*, Miguel Inacio da Silva Filho[1], Michael Fuchs[3], Sabine Ponader[3], Elke Pogge von Strandmann[3], Lewin Eisele[6], Stefan Herms[4,5], Per Hofmann[4,5], Andreas Engert[3], Kari Hemminki[1,2], Asta Försti[1,2]

[1]German Cancer Research Center (DKFZ), Division of Molecular Genetic Epidemiology, Heidelberg, 69120, Germany

[2]Center for Primary Health Care Research, Lund University, Malmö, 20502, Sweden

[3]Department of Internal Medicine I, University Hospital of Cologne, Cologne, 50924, Germany

[4]Institute of Human Genetics and Department of Genomics, University of Bonn, 53127, Germany

[5]Department of Biomedicine, Division of Medical Genetics, Basel, University of Basel, 4058, Switzerland

[6]Institute for Medical Informatics, Biometry and Epidemiology, University Hospital Essen, University Duisburg-Essen, Essen, 45122,Germany

Table A. Association between homozygosity and susceptibility to HL for individual SNPs

SNP	CHR	BP$	Cases homozyg.	Cases heterozyg.	Controls homozyg.	Controls heterozyg.	chi2	*P**	*q*†
rs11757571	6	31540765	755	151	1100	117	22.78	1.81-06	0.23
rs9807444	18	12290936	690	216	1027	189	22.61	1.97-06	0.23
rs11067657	12	114486773	731	175	1073	144	22.19	2.46-06	0.23
rs4797673	18	12306492	691	215	1028	189	22.13	2.53-06	0.23
rs10472114	5	59071314	497	409	791	426	21.95	2.79-06	0.23
rs11080567	18	12302075	691	215	1027	190	21.65	3.26-06	0.23
rs1430416	3	65926792	395	510	655	562	21.08	4.38-06	0.24
rs11752262	6	31539735	741	165	1081	136	20.56	5.77-06	0.24
rs3830135	6	32656441	778	127	951	266	20.53	5.84-06	0.24
rs910873	20	32635432	827	79	1030	187	20.32	6.51-06	0.24
rs17305573	20	32643813	826	79	1029	187	20.31	6.58-06	0.24
rs10484564	6	32860026	684	221	1016	200	20.23	6.86-06	0.24

$ Genome build hg18

* *P* was calculated using a simple 2x2 chi^2 test based on the number of homozygotes and heterozygotes at each SNP in cases and controls.

† *q* values representing the false discovery rate (FDR)

Table A shows a test performed for any association between homozygosity (whether for the major or the minor allele) and the susceptibility to HL on a SNP-by-SNP basis in our sample series. Results for the best SNPs with P < $1*10^{-5}$ are shown.

Table B. Association between F_{ROH} and HL

	F_{ROH} from all ROH				
**F_{ROH}**	**Cases**	**Controls**	**OR**	**95% CI**	***P***
<5-03	248	283	1.00	Ref.	
5-03-7-03	227	304	0.85	0.66-1.08	0.19
7-03-9-03	223	308	0.82	0.64-1.05	0.12
>9-03	208	322	0.73	0.57-0.94	0.01
	F_{ROH} <0.007 (below median)				
**F_{ROH}**	**Cases**	**Controls**	**OR**	**95% CI**	***P***
<1-06	243	288	1.00	Ref.	
2-06-3-06	240	291	0.97	0.76-1.24	0.85
3-06-4-06	214	317	0.80	0.62-1.02	0.07
>4-06	209	321	0.77	0.60-0.98	0.03
	F_{ROH} >0.007 (above median)				
**F_{ROH}**	**Cases**	**Controls**	**OR**	**95% CI**	***P***
<1-06	241	290	1.00	Ref.	
2-06-3-06	220	311	0.85	0.66-1.08	0.19
4-06-5-06	216	315	0.82	0.64-1.05	0.12
>5-06	229	301	0.91	0.71-1.16	0.47

Table B shows an overview of the association between homozygosity and the susceptibility to HL by comparing the number of cases against equally distributed numbers of controls for different F_{ROH} values.

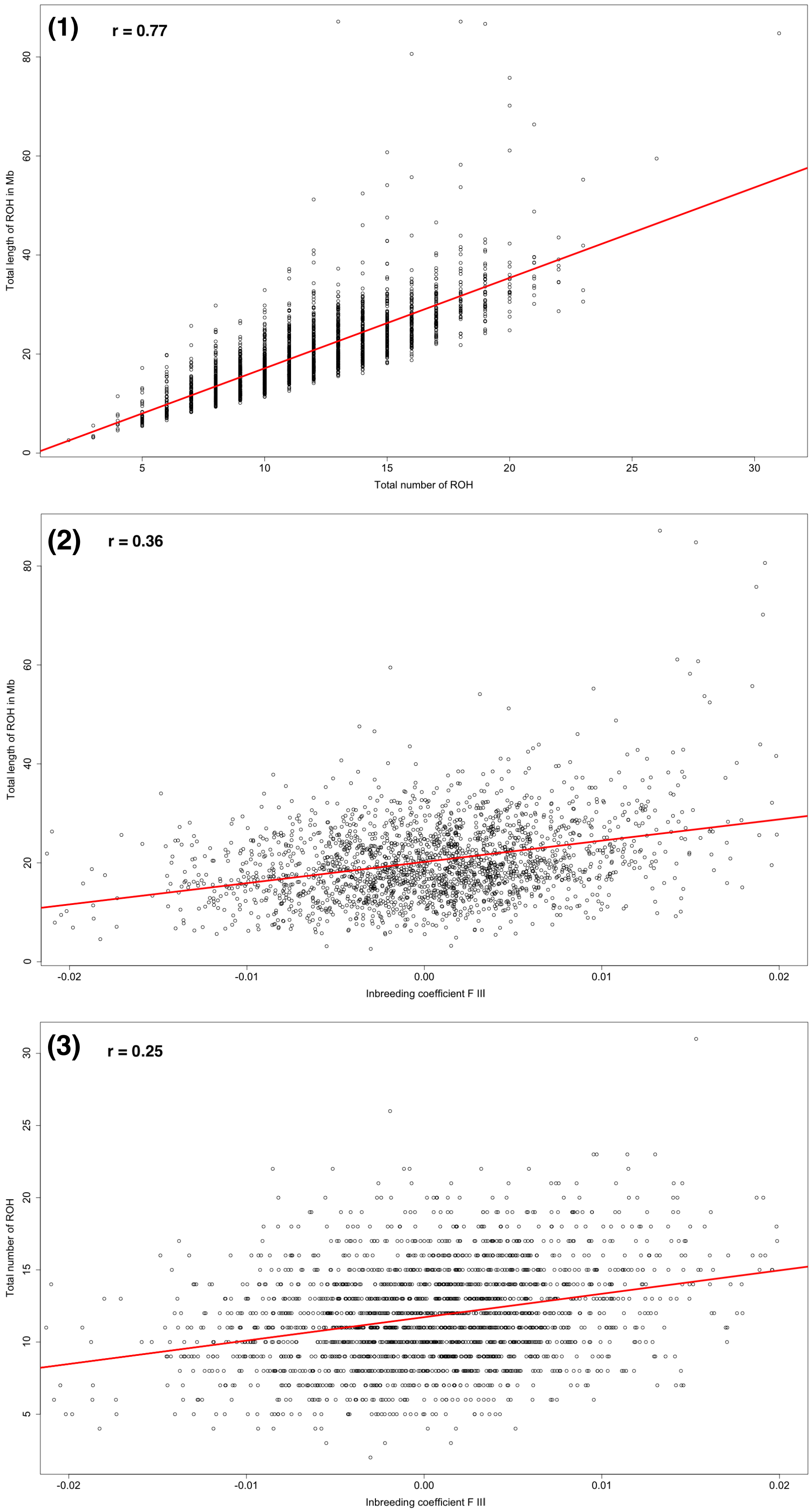
(1)
r = 0.77
Total length of ROH in Mb
Total number of ROH
(2)
r = 0.36
Total length of ROH in Mb
Inbreeding coefficient F III
(3)
r = 0.25
Total number of ROH
Inbreeding coefficient F III

Fig A. Pearson's correlation coefficients for different consanguinity measures

The total length of individual ROHs is highly correlated with the total number of ROHs per individual (r=0.77, $P < 2.20*10^{-16}$) (1). A moderate association is determined for the total length of ROHs per individual and the individual inbreeding coefficient F III (r=0.36, $P < 2.20*10^{-16}$) (2), while the lowest association was determined for the total number of ROHs per individual and the individual inbreeding coefficient F III (r=0.25, $P < 2.20*10^{-16}$) (3).

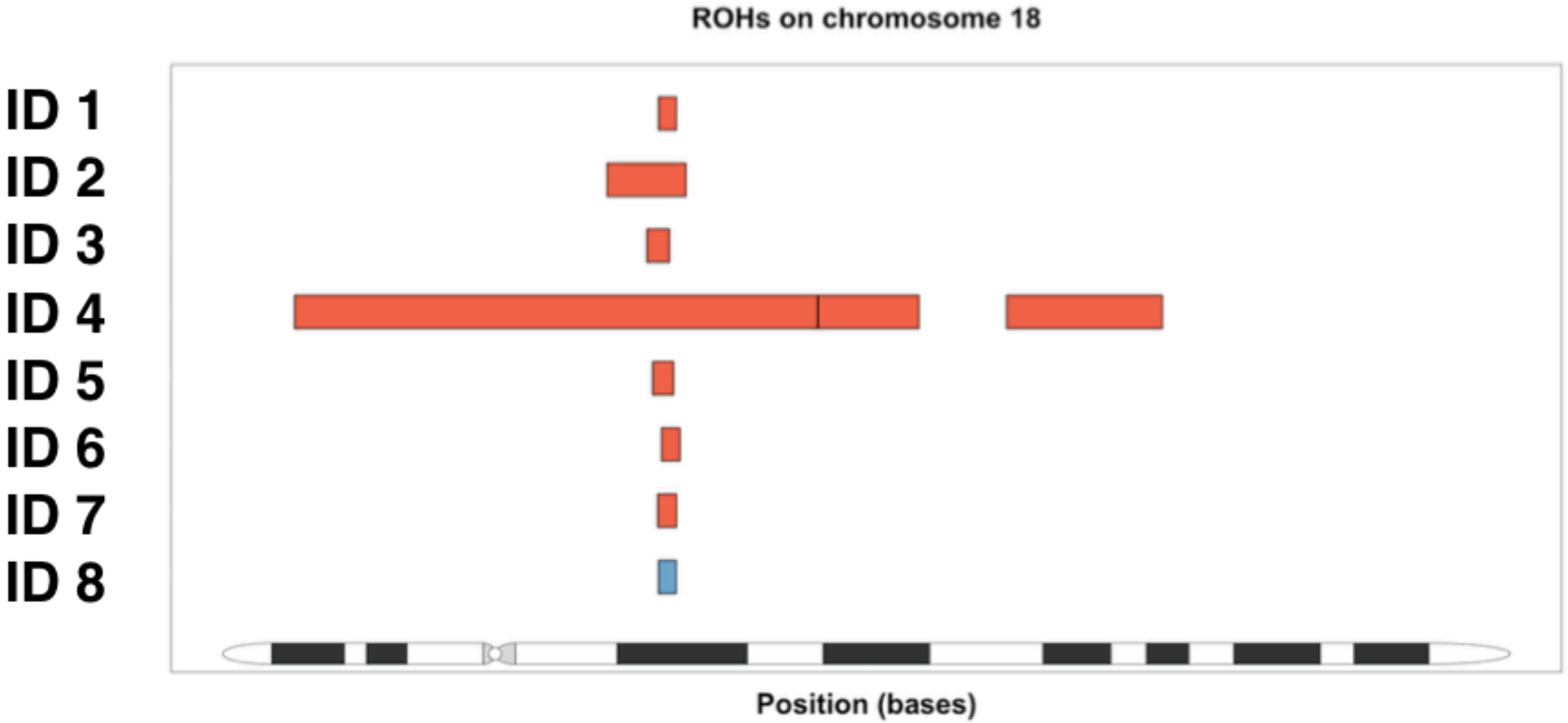

Fig B. ROHs on chromosome 18. The figure shows an example of overlapping ROH regions on chromosome 18 (ROH1 in Table 3 of the manuscript).
